# Role of the CXCL13/CXCR5 Axis in Autoimmune Diseases

**DOI:** 10.3389/fimmu.2022.850998

**Published:** 2022-03-04

**Authors:** Zijian Pan, Tong Zhu, Yanjun Liu, Nannan Zhang

**Affiliations:** ^1^ National Center for Birth Defect Monitoring, Key Laboratory of Birth Defects and Related Diseases of Women and Children, Ministry of Education, West China Second University Hospital, and State Key Laboratory of Oral Diseases, Sichuan University, Chengdu, China; ^2^ West China Hospital of Stomatology, Sichuan University, Chengdu, China

**Keywords:** CXCL13, CXCR5, chemokine, autoimmunity, therapeutic target

## Abstract

CXCL13 is a B-cell chemokine produced mainly by mesenchymal lymphoid tissue organizer cells, follicular dendritic cells, and human T follicular helper cells. By binding to its receptor, CXCR5, CXCL13 plays an important role in lymphoid neogenesis, lymphoid organization, and immune responses. Recent studies have found that CXCL13 and its receptor CXCR5 are implicated in the pathogenesis of several autoimmune diseases, such as rheumatoid arthritis, multiple sclerosis, systemic lupus erythematosus, primary Sjögren’s syndrome, myasthenia gravis, and inflammatory bowel disease. In this review, we discuss the biological features of CXCL13 and CXCR5 and the recent findings on the pathogenic roles of the CXCL13/CXCR5 axis in autoimmune diseases. Furthermore, we discuss the potential role of CXCL13 as a disease biomarker and therapeutic target in autoimmune diseases.

## Introduction

Chemokines are a family of small chemotactic cytokines that play roles in immune cell trafficking (1). Chemokines can be divided into four subfamilies depending on the position of the first two conserved N-terminal cysteine residues: XC, CC, CXC, and CX3C chemokines (2). By interacting with their corresponding chemokine receptors, seven transmembrane G protein-coupled receptors (GPCRs), chemokines play multiple roles in immune response, embryonic development, and angiogenesis (3). In addition, aberrant activation of chemokine signaling pathways is implicated in the development of many human diseases, such as autoimmune diseases, chronic inflammatory diseases and cancers (2).

CXCL13 is initially known as B cell-attracting chemokine 1 (BCA-1) and B-lymphocyte chemoattractant (BLC), for its strong chemotaxis to B cells (4, 5). CXCL13 is constitutively expressed in the B-cell follicles of secondary lymphoid organs (5, 6). Follicular dendritic cells (FDCs) have been proved to be an important source of CXCL13 (7). In addition, CXCL13 is also produced by T follicular helper (Tfh) cells in humans, but not by murine Tfh cells (8). Tfh cells, with the expression of CXCR5, B cell lymphoma 6 (BCL-6), interleukin-21 (IL-21), programmed death-1 (PD-1), and inducible T cell co-stimulator (ICOS), dwell in the germinal centers (GCs) of secondary lymphoid organs where these cells can support B-cell survival and differentiation with the help of CD40L/CD40 interactions and cytokines such as interleukin-4 (IL-4) and IL-21 (9). When interacting with B cells, Tfh cells can release dopamine, which can promote the rapid translocation of intracellular ICOSL to cell surface of B cells (10). The ICOSL/ICOS interaction can further augment the accumulation of CD40L and chromogranin B granules at the synapse of Tfh cells, facilitating T-B interactions and GCs development (10).

Besides, expression of CXCL13 is also detected in CD4^+^ PD-1^hi^ CXCR5^-^ T peripheral helper (Tph) cells in RA synovial tissue specimens (11). The production of CXCL13 by Tph cells could exert an indispensable role in recruiting CXCR5^+^ B cells (11, 12). Due to the lack of expression of Th-related cytokines such as interferon-γ (IFN-γ), IL-4, and IL-17, the Tph cells are recognized as a distinct Th-cell subpopulation from Th1 cells, Th2 cells, and Th17 cells (13). Besides CXCL13 secretion, these cells also express B lymphocyte-induced maturation protein-1 (BLIMP-1), IL-21, ICOS, and MAF to further promote B-cell activation and differentiation (11, 14). And the transcription factor Sox-4 is responsible for CXCL13 production in Tph cells (15). Compared with Tfh cells, Tph cells do not express CXCR5, however, these cells can express other chemokine receptors such as CCR2, CCR5, and CX3CR1, which may determine their peripheral localization (11). Although Tfh cells and Tph cells can both exert B-cell helper activities, the B-cell helper abilities between the two cells to different B-cell subpopulations are distinct (16). For instance, Tfh cells could help both naïve B cells and memory B cells, while the targets of Tph cells are mainly limited to memory B cells (16).

CXCR5, formerly named Burkitt’s lymphoma receptor 1 (BLR1), is the sole receptor of CXCL13 (4, 5). Physically, CXCR5 is highly expressed on mature B cells, subpopulations of CD4^+^ T cells [i.e. Tfh and T follicular regulatory (Tfr) cells], and skin-derived migratory dendritic cells (DCs) (17–20). By attracting these cells in a CXCR5-dependent manner, CXCL13 is essential in the development, organization, and immune activation of secondary lymphoid organs including lymph nodes (LNs), Peyer’s patches, and spleen (21). Additionally, CXCL13/CXCR5 axis is also required for B1 cells homeostasis (22) and B-cell receptor (BCR)-induced B-cell activation (23).

**Figure 1:**
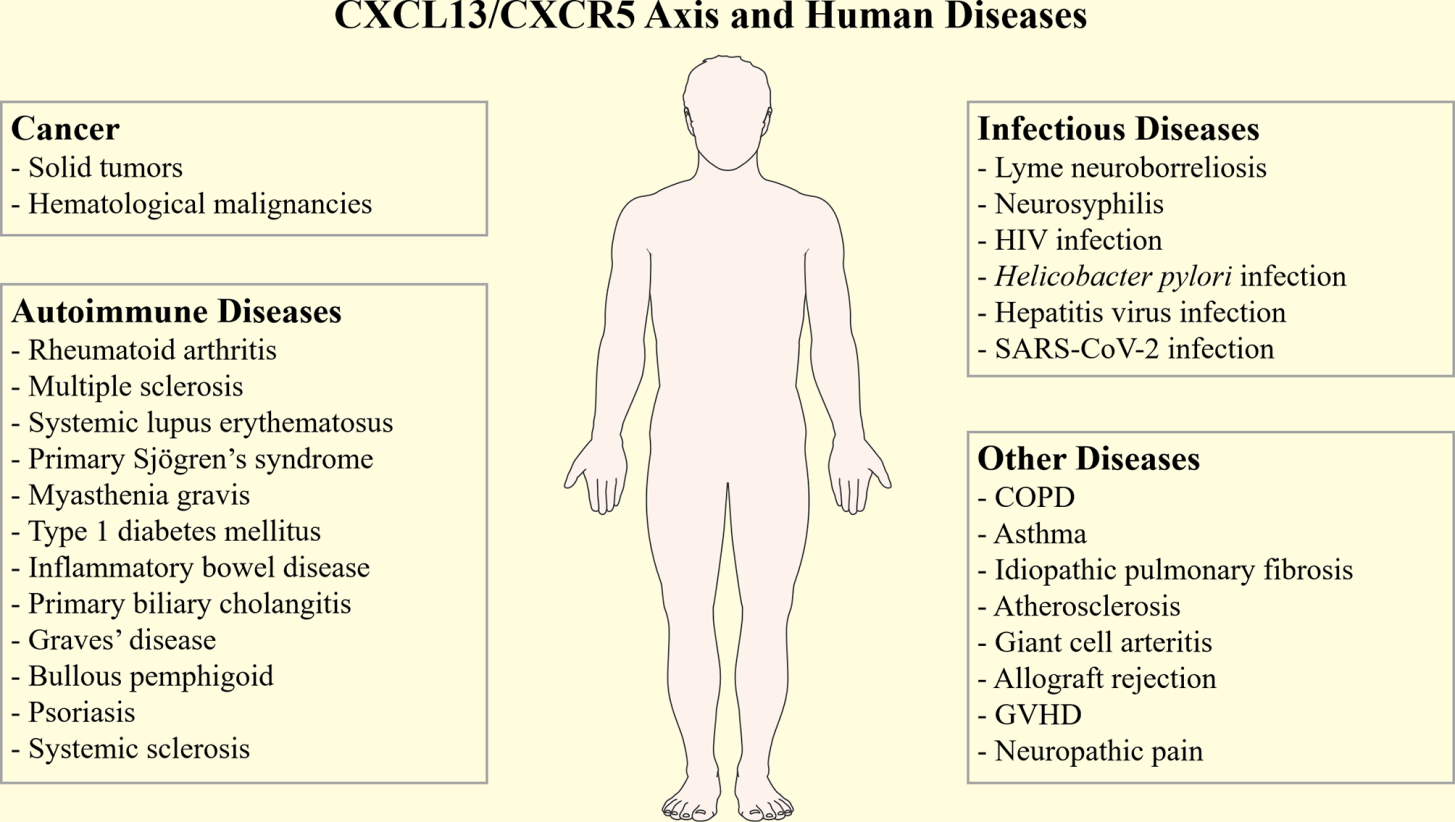
The CXCL13/CXCR5 axis in human diseases. The CXCL13/CXCR5 axis participates in the pathogenesis and progression of many human diseases, such as cancers, autoimmune diseases, infectious diseases, idiopathic pulmonary fibrosis, transplantation rejection, and neuropathic pain. COPD, chronic obstructive pulmonary disease; GVHD, graft versus host disease; SARS-CoV-2, severe acute respiratory syndrome coronavirus-2.

Overall, the CXCL13/CXCR5 axis can affect the B-cell and Tfh cells’ functions, and plays important roles in immunity. Dysregulated CXCL13 expression has been associated with various human diseases, such as cancers (24), infectious diseases (25, 26), idiopathic pulmonary fibrosis (IPF) (27, 28), transplantation rejection (29), and neuropathic pain (30) (**Figure 1**). Moreover, CXCL13 is also related to many autoimmune diseases, such as rheumatoid arthritis (RA), multiple sclerosis (MS), systematic lupus erythematosis (SLE), primary Sjögren’s syndrome (pSS), myasthenia gravis (MG), and inflammatory bowel diseases (IBD). In this review, we provide a comprehensive overview of the pathogenic role of CXCL13/CXCR5 axis in autoimmune diseases, while also discussing the potential usage of CXCL13 as a novel clinical biomarker and treatment target.

## Biological Functions of the CXCL13/CXCR5 Axis

The CXCL13/CXCR5 axis plays an essential role in the neogenesis and organization of secondary lymphoid tissues, and in immune responses (31). The role of the CXCL13/CXCR5 axis in the development of secondary lymphoid organs has been documented in CXCL13- and CXCR5-deficient mice, whose lymphoid organs exhibit impaired development both in size and organization (32, 33). In CXCL13^-/-^ and CXCR5^-/-^ mice, most of the LNs (e.g., inguinal, iliac, sacral, brachial, and axillary LNs) are absent, consistent with impaired numbers of Peyer’s patches (33). However, facial, superficial cervical, and mesenteric LNs have been confirmed to be retained in these mice (33). In the fetal stage, CXCL13 produced by mesenchymal lymphoid tissue organizer (mLTo) cells attracts lymphoid tissue inducer (LTi) cells towards the parenchyma of the LNs anlagen (34, 35). Recruited LTi cells subsequently upregulate lymphotoxin-α1β2 (LTα1β2) expression and activate mLTo cells through LTα1β2-lymphotoxin β receptor (LTβR) interactions (36). Then, activated mLTo cells enhance the expression of CXCL13 together with other homeostatic chemokines (e.g. CCL19 and CCL21), cytokines [e.g. IL-7 and receptor activator of nuclear factor-κB ligand (RANKL)], and adhesion molecules [e.g. vascular cell adhesin molecule 1 (VCAM-1) and intercellular adhesion molecule 1 (ICAM-1)] to further recruit and retain more LTi cells, thus forming a positive feed-forward loop that contributes to LNs formation (37).

In addition, CXCL13 and CXCR5 are required for B-cell homing in secondary lymphoid organs (33). B cells can migrate toward B cell follicles following the CXCL13 gradients produced by FDCs, the constitutive stromal cells in the follicles (38), while in the CXCR5^-/-^ mice, B cells could not migrate toward the follicles of secondary lymphoid organs (33). Furthermore, CXCL13 induces recruited B cells to upregulate LTα1β2, which can promote FDCs development and increase the expression of CXCL13, leading to a positive feedback loop which is crucial for the development of B cell follicles (33).

CXCL13 is also involved in GCs compartmentalization, an essential biological process for adaptive immune responses (23, 39). CXCL13 produced by FDCs also attracts B cells toward the light zones within GCs, where B cells undergo affinity selection and become long-lived plasma cells or memory B cells (37, 39). The CXCL13/CXCR5 axis also regulates T-cell positioning, and thus facilitates GCs response (40). CXCL13 can attract the Tfh cells toward the boundary between T cell zones and B cell follicles (T-B border), where Tfh cells can interact with activated B cells and further promote the B-cell activation and proliferation (41). Indeed, in mice with T cells lacking CXCR5, the GCs response is impaired, as demonstrated by the fewer and smaller GCs and reduced B-cell frequency found within GCs (42). CXCL13/CXCR5 axis also regulates the migration of Tfr cells, which can suppress excessive humoral responses by acting on mature B cells and Tfh cells within GCs (19).

The CXCL13/CXCR5 axis is also implicated in B1 cell response (22). CXCL13 is constitutively produced by peritoneal macrophages, and attracts B1 cell toward the peritoneal cavity, which is important in maintaining the body cavity innate immunity (22). Bröker et al. found that toll-like receptor 2 (TLR2) activation consistent with TLR2-triggered IL-10 could induce the production of CXCL13 and C5a in peritoneal macrophages (43). They further found that the C5a could subsequently enhance CXCL13 secretion of peritoneal macrophages by interacting with C5a receptor (C5aR) (43).

**Figure 2:**
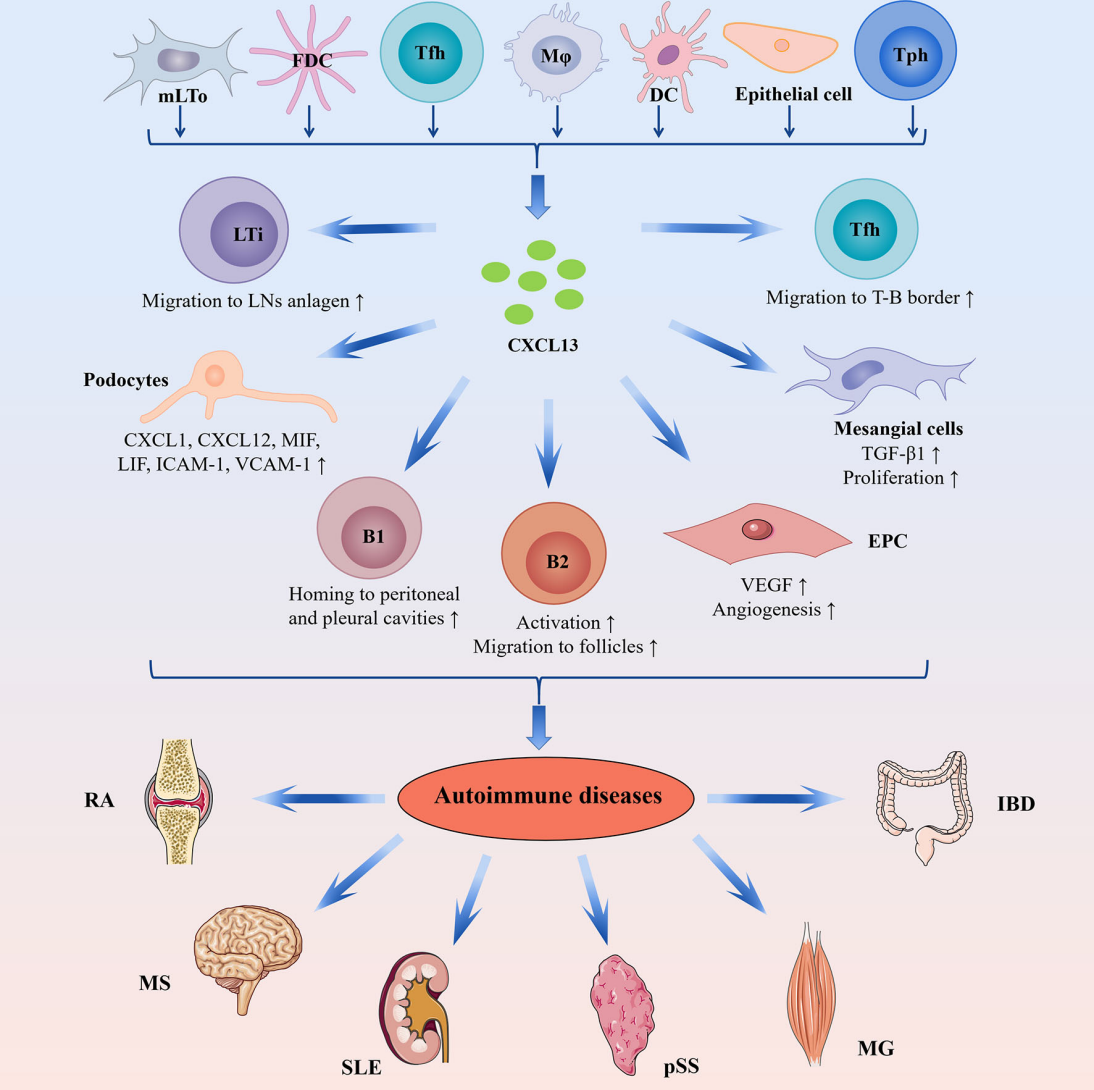
The complicated role of CXCL13-mediated immune responses in autoimmune diseases. CXCL13 can be produced by mLTo cells, FDC, Tfh cells, macrophages, DCs, epithelial cells, and Tph cells. In the fetal stage, CXCL13 promotes migration of LTi cells toward the parenchyma of the LNs anlagen, where LTi cells interact with mLTo cells to induce lymphoid neogenesis. CXCL13 is involved in the lymphoid organization by attracting B cells toward B-cell follicles. During an immune response, CXCL13 attracts B cells toward the light zones of GCs, where B cells undergo affinity selection and become long-lived plasma cells or memory B cells. Furthermore, CXCL13-mediated Tfh cells migration is essential in facilitating GCs response. In addition, CXCL13/CXCR5 axis is also implicated in B1 cell response through attracting B1 cell toward the body cavity. In autoimmune diseases, ectopic CXCL13 expression promotes ectopic lymphoid neogenesis and the production of disease-specific autoantibodies. In RA, CXCL13 drives EPC homing and VEGF expression, thus inducing angiogenesis in synovial tissue. In LN, CXCL13 promotes proliferation and TGF-β1 production of mesangial cells and induces podocyte secretion of proinflammatory cytokines/chemokines such as CXCL1, CXCL12, MIF, LIF, and soluble ICAM-1 and VCAM-1. DCs, dendritic cells; EPC, endothelial progenitor cells; FDC, follicular dendritic cells; GCs, germinal centers; IBD, inflammatory bowel disease; ICAM-1, intercellular adhesion molecule 1; LN, lupus nephritis; LIF, leucocyte inhibitory factor; LTi, lymphoid tissue inducer; MG, myasthenia gravis; MIF, macrophage inhibitory factor; mLTo, mesenchymal lymphoid tissue organizer; MS, multiple sclerosis; pSS, primary Sjögren’s syndrome; RA, rheumatoid arthritis; SLE, systemic lupus erythematosus; Tfh, T follicular helper; TGF-β1, transforming growth factor β1; Tph, T peripheral helper; VCAM-1, vascular cell adhesion molecule 1; VEGF, vascular endothelial growth factor.

CXCL13/CXCR5 axis is also involved in BCR-triggered B-cell activation by shaping cell dynamics, which enhances antigen gathering at the immune synapse (23). The possible mechanisms are that CXCL13/CXCR5 axis promotes the membrane ruffling and adhesion supported by lymphocyte function associated antigen 1 (LFA-1) and integrates the BCR signaling activation by the migratory junction supported by LFA-1 (23). In a word, CXCL13/CXCR5 axis is important in regulating B-cell homeostasis (**Figure 2**).

## CXCL13/CXCR5 Protein Structure

**Figure 3:**
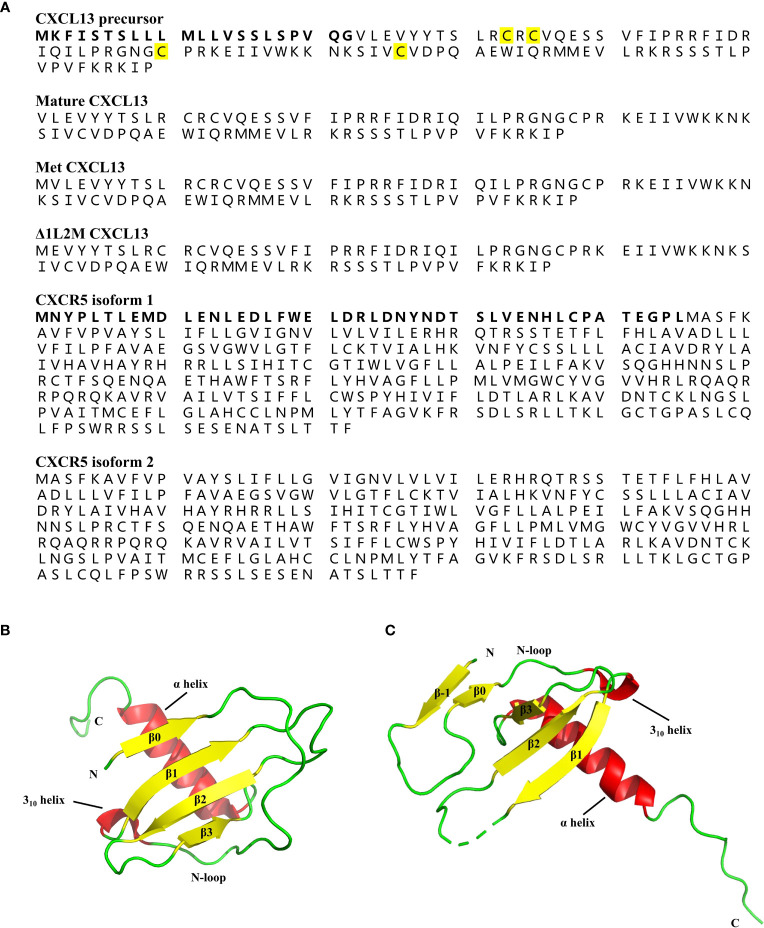
Protein structures of CXCL13 and CXCR5. **(A)** The amino acid sequence of human CXCL13 precursor, mature CXCL13, Met CXCL13, Δ1L2M CXCL13, and two isoforms of human CXCR5. The human CXCL13 protein consists of 109 amino acids, including a signal peptide of 22 amino acids (marked in bold). The four conserved cysteine residues are marked in yellow. The human CXCR5 has two isoforms due to the alternatively spliced transcript variants. The difference between the two isoform of CXCR5 is additionally marked in bold. **(B)** The tertiary structure of Met CXCL13 [Protein Data Bank (PDB) ID: 7JNY]. The N-terminus of Met CXCL13 forms a β0-sheet, followed by a long N-loop ending in a short 3_10_-helix, and the central three-stranded anti-parallel β-sheet, and a C-terminal α-helix. β-sheet is indicated by yellow arrows, and α-helix and 3_10_-helix are indicated by red cylinders. **(C)** The tertiary structure of Δ1L2M CXCL13 monomer [PDB ID: 6VGJ]. In Δ1L2M CXCL13, the N-terminus folds into a β-strand (β-1), followed by a β0-sheet, and a canonical chemokine core domain. β-sheet is indicated by yellow arrows, and α-helix and 3_10_-helix are indicated by red cylinders.

CXCL13 is a 109-amino-acid protein with a signal peptide of 22 amino acids, containing four cysteine residues showing a C-X-C chemokine pattern (**Figure 3A**) (4). Typically, the tertiary structure of chemokines is relatively conserved, consisting of a disordered N-terminal ‘signaling domain’, followed by a ‘core domain’, which includes an ‘N-loop’, a 310-helix, a three-stranded b-sheet, and a C-terminal a-helix (44). Although the crystal structure of wildtype (WT) human CXCL13 protein alone has not been solved yet, Tu et al. crystallized two structures of WT human CXCL13 in complex with antibody single chain variable fragments (scFvs) (45). Moreover, Rosenberg et al. solved the structures of two engineered CXCL13 mutants, i.e. Met CXCL13 (mature CXCL13 protein contains a N-terminal initiating methionine) and D1L2M CXCL13 (mature CXCL13 protein in which Val1 is deleted and Leu2 is replaced by Met) (196). In Met CXCL13, the N-terminus forms an additional parallel bstrand (b0), which interacts with the core b-sheet, thus forming a four-stranded b-sheet (**Figure 3B**). A b-strand (b-1) in the N-terminus is also evident in D1L2M CXCL13 (**Figure 3C**), and it interacts with both the core b-sheet from an adjacent monomer and a b-strand (b0) formed by the N-loop in the same monomer. These structures, together with the structures of CXCL13 in complex with scFvs demonstrate a flexible N-terminus, as well as a highly disordered C-terminal extension, but a fairly rigid canonical chemokine core domain of CXCL13.

The human CXCR5 is a seven transmembrane GPCR, and it contains two isoforms due to the alternatively spliced transcript variants (47). Compared with the predominant transcript variant 1, the transcript variant 2 is different in the 5’ end, resulting in translation starting from the downstream in-frame AUG, leading to an isoform with a shorter N-terminus (**Figure 3A**) (47). However, the crystal structure of CXCR5 is largely unknown. Rosenberg et al. explored the effects of N-terminal length variation and side-chain composition of CXCL13 on CXCR5 activity (45). They found that the orthosteric pocket of CXCR5 can tolerate minor variations in the length and side-chain of the CXCL13 N-terminus, without severely impairing the activity (45). And the enlarged bulk in the CXCL13 orthosteric site was well tolerated by CXCR5, while a loss of contacts between CXCL13 and CXCR5 was less tolerated (45). However, the structural basis for CXCL13-CXCR5 interactions is also less studied.

## CXCL13/CXCR5-Mediated Signaling Pathways

CXCL13 binding to CXCR5, the seven-transmembrane GPCR, induces a variety of downstream signaling pathways, leading to various cellular events such as migration, survival, proliferation, and modulation of gene transcription (24). The signal transduction pathways mediated by CXCR5 follow the classical GPCR activation pattern (24). G proteins are heterotrimers that comprise three subunits: Gα, Gβ, and Gγ (48). Ligand binding leads to a three-dimensional conformation change of GPCRs, and results in the dissociation of Gα subunit from Gβγ dimer, which then regulates the downstream effectors (48). However, the specific transduction mechanisms involved in CXCL13-CXCR5 signaling may vary in a cell- and stimulus-dependent manner (24).

Early studies demonstrated that CXCR5 induced Ca^2+^ influx and chemotaxis independently of inhibitory G proteins, whereas CXCR5-mediated mitogen-activated protein kinase (MAPK) signaling *via* extracellular signal-regulated kinase 1 and 2 (ERK1/2) was found to occur in an inhibitory G proteins dependent manner (49). Structurally, these signaling pathways induced by CXCR5 are dependent on the second intracellular domain of CXCR5 (49). Moreover, Barroso et al. found that these pathways also depended on the regulatory role of Epstein-Barr virus-induced G-protein coupled receptor 2 (EBI2) on CXCR5 (50). Through forming a heterodimer with CXCR5, EBI2 modulates CXCL13/CXCR5-induced cell responses (50).

**Figure 4:**
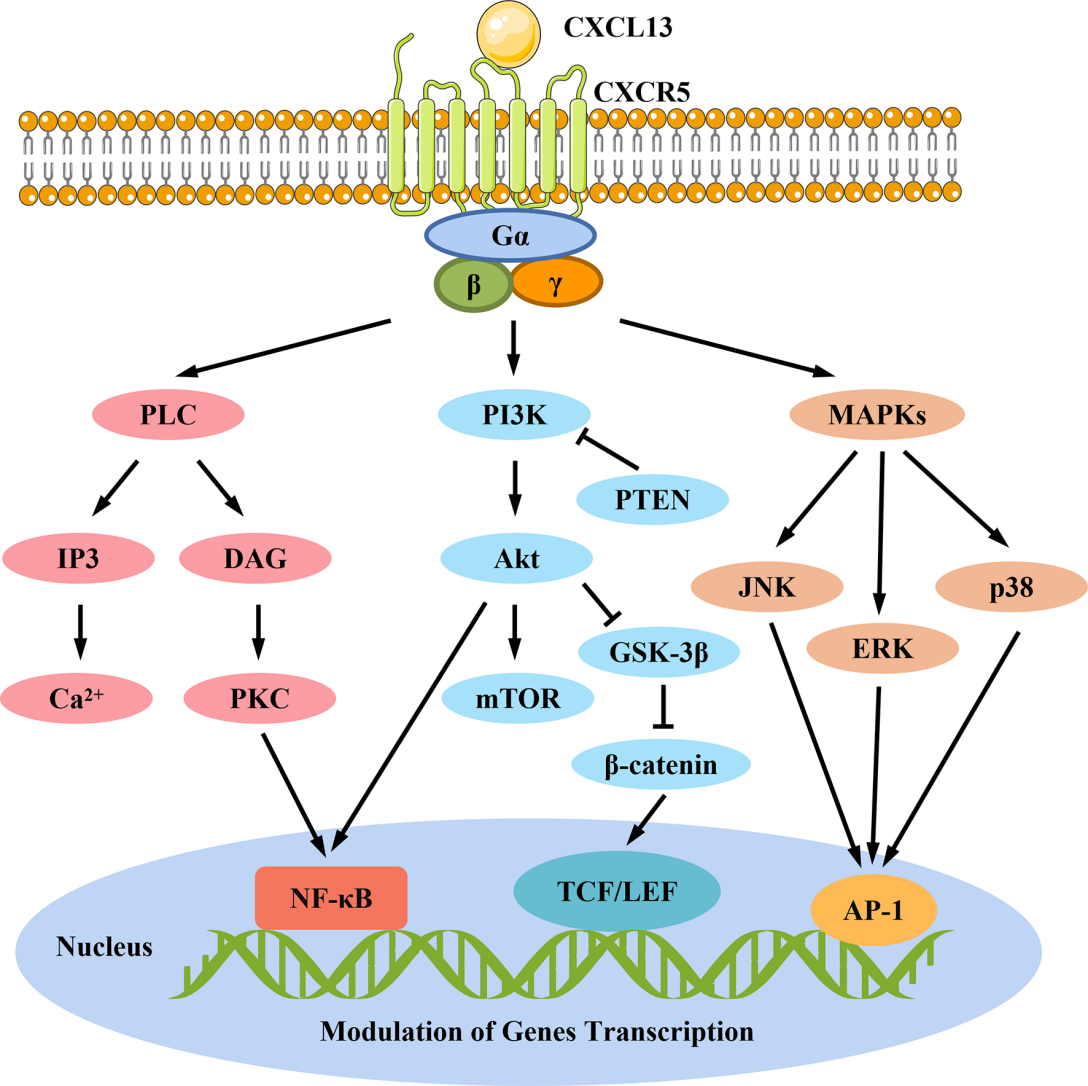
CXCL13/CXCR5-mediated signaling pathways. CXCL13 exerts its biological functions through activating CXCR5, a chemokine receptor coupled to G-protein heterotrimer. Upon activation, CXCR5 undergoes conformation change and induces the cycle of G-protein activation, leading to a cascade of downstream signal transduction pathways including: (1) activation of PLC leads to conversion of PIP2 to IP3 and DAG. IP3 can promote the release of Ca^2+^ from intracellular stores into the cytoplasm. On the other hand, DAG consistent with increased Ca^2+^ activate PKC, which contributes to the activation of transcription factor NF-κB to promote cell migration; (2) activation of PI3K can trigger the activation of Akt, thus stimulating downstream NF-κB, mTOR, and GSK-3β/β-catenin/TCF/LEF signaling, which play key roles in tumor cell growth, proliferation, invasion, and migration; (3) CXCR5 also activates MAPK pathways utilizing JNK, ERK, and p38 *via* G-protein, which may further stimulate AP-1 to promote cell proliferation and inflammation. AP-1, activating protein-1; DAG, diacylglicerol; ERK, extracellular signal-regulated kinase; GSK-3β, glycogen synthase kinase-3β; IP3, inositol triphosphate; JNK, c-Jun N-terminal kinase; mTOR, mammalian target of rapamycin; NF-κB, nuclear factor-κB; PI3K, phosphoinositide 3-kinase; PKC, protein kinase C, PLC, phospholipase C; PTEN, phosphatase and tensin homologue; TCF/LEF, T-cell factor/lymphoid enhancer-binding factor.

Emerging studies have also shown that dysregulated CXCR5-mediated pathways are associated with human diseases, especially with tumors. For instance, in human osteosarcoma cell lines, it is reported that CXCL13/CXCR5 signaling relies on phospholipase C-β (PLCβ) and protein kinase C-α (PKCα) to activate downstream nuclear factor-κB (NF-κB) signals, thereby promoting cell migration (51). In return, PKCϵ overexpression and phosphatase and tensin homologue (PTEN) inactivation were found to upregulate CXCL13 expression through the non-canonical NF-κB pathway, resulting in prostate cancer cell migration and tumorigenesis (52). The CXCR5-mediated pathway also utilizes phosphoinositide 3-kinase (PI3K) to activate Akt, resulting in the activation of downstream NF-κB and mammalian target of rapamycin (mTOR) pathways involved in cell growth and invasion (52, 53). Recent studies have found that CXCR5 regulates downstream glycogen synthase kinase-3β (GSK-3β) and β-catenin through the PI3K-Akt pathway, leading to epithelial cell proliferation and tumorigenesis through upregulating cyclin D1 and c-myc expressions (54). In addition to the ERK1/2 pathway, CXCR5 also activates the MAPK pathway utilizing c-Jun N-terminal kinase (JNK) and p38, to mediate prostate cancer cell proliferation and inflammatory pain, respectively (55, 56). The signaling pathways for CXCL13/CXCR5 axis described above are shown in **Figure 4**.

## The CXCL13/CXCR5 Axis in Autoimmune Diseases

Autoimmune diseases are characterized by the presence of autoantibodies or autoreactive T cells that attack self-antigens, and thus lead to damage and dysfunction of tissues and organs (57). Autoimmune diseases comprise a large spectrum of diseases such as RA, SLE, MS, pSS, MG, and IBD etc. (58). Chemokines play important roles in regulating immune responses and mediating inflammation, as well as being involved in the pathogenesis of autoimmune diseases (2). For instance, CXCL9, CXCL10, and CXCL11 promote Th1 polarization and mediate Th1 responses in autoimmune diseases (59).

CXCL13 is vital in the lymphoid neogenesis, maintaining the architecture of secondary lymphoid tissues, and immune responses (24). CXCL13 is also involved in the initiation and organization of ectopic lymphoid-like structures (ELSs), the organized lymphocyte aggregates developed at sites of inflammation in target tissues of autoimmune diseases (60). ELSs are dynamic structures resembling secondary lymphoid organs in both histological structures and gene expression profile (60). In ELSs of inflammatory tissues, elevated expression of CXCL13 can regulate B-cell infiltration and positioning, and can also regulate B-cell shuttle inside the ectopic GCs (61). In addition, Tfh cells can also be attracted by CXCL13 towards the proximity of B cells in ELS, and further exert B-cell help (62). ELSs can serve as functional GCs that promote autoreactive B-cell response and increase the local autoantibody production (62).

The pivotal role CXCL13/CXCR5 axis in ELSs development has been detected in several autoimmune diseases such as RA and pSS. In RA, mRNA and protein levels of CXCL13 are increased in ectopic lymphoid follicles in the synovial tissues, especially in the area with B cells accumulation (63, 64). In addition, the mRNA expression levels of CXCL13 are positively correlated with ELSs development in inflamed synovial tissues of RA patients (65). These studies indicate that CXCL13 may attract B cells and contribute to the formation ELSs in chronic arthritis (65). CXCL13 is overexpressed in ELSs in salivary glands of patients with pSS (66). In salivary gland tissues of pSS patients, CXCL13 expression is associated with increased size of lymphoid aggregates and the progressive organization lymphoid-like structures (67). The ELSs in pSS can further promote B-cell expansion, local antibody production, and lymphomagenesis (68). Besides, CXCL13/CXCR5 axis plays similar roles in other autoimmune diseases, such as MS (69), SLE (70), MG (71), and so on. Hence, CXCL13 may affect the pathogenesis of autoimmune diseases through regulating ELSs development (68). In addition, the CXCL13/CXCR5 axis may also have disease-specific pathogenetic mechanisms in certain autoimmune diseases, which will be discussed further below. (**Figure 2** and **Table 1**).

**Table 1:** Expression levels and effects of CXCL13/CXCR5 axis in autoimmune diseases.

Disease	Expression levels	Effects	Reference
RA	Increased CXCL13 in serum, plasma, synovial tissues, and synovial fluids.	Promotes ectopic lymphoid neogenesis. Modulates B10^+^ cells attraction and activation. Promotes EPC homing and angiogenesis.	(63, 72–78)
MS	Elevated CXCL13 in serum, plasma, CSF, and active MS lesions.	Promotes ectopic lymphoid neogenesis.	(77, 79–82)
SLE	Increased CXCL13 levels in serum. Increased CXCL13 and CXCR5 expressions in renal cortex of LN patients.	Regulates B-cell and double-negative T cells trafficking. Promotes mesangial cells proliferation and TGF-β1 production. Induces proinflammatory cytokines and chemokines secretion of podocytes.	(83–88)
pSS	Increased CXCL13 in serum, saliva, salivary glands tissues, and salivary gland secretome. Elevated CXCR5^+^ cells accumulate within focal infiltrates of minor salivary glands.	Promotes CXCR5^+^ cells recruitment and ectopic lymphoid neogenesis.	(89–93)
MG	Increased CXCL13 in serum and thymus.	Promotes ectopic GCs formation in thymus.	(71, 94–98)
T1DM	Increased CXCL13 and CXCR5 expression in NOD mice.	Promotes ectopic lymphoid neogenesis.	(99, 100)
IBD	Increased CXCL13 in serum. Increased CXCL13 and CXCR5 expression within lymphoid aggregates in lesions.	Promotes CD4^+^ CXCR5^+^ T cells migration.	(101–103)
PBC	Increased CXCL13 expression in liver tissue. Increased frequencies of CXCR5^+^ CD4^+^ T cells in portal tracts.	Promotes CXCR5^+^ cells recruitment.	(104)
GD	Increased CXCL13 and CXCR5 expression in thyroid tissue.	Promotes CXCR5^+^ cells recruitment and ectopic lymphoid neogenesis.	(105–107)
BP	Increased CXCL13 in serum. Increased CXCL13^+^ cells and CXCR5^+^ cells in lesional skin.	Not fully clarified.	(108)
Psoriasis	Increased CXCL13 in serum, plasma, and cutaneous lesions.	Not fully clarified.	(109, 110)
SSc	Increased CXCL13 in serum and lesional skin.	Promotes B-cell responses.	(111)
AIP	Increased CXCL13 expression in pancreatic tissues	Not fully clarified.	(112)

AIP, autoimmune pancreatitis; BP, bullous pemphigoid; CSF, cerebrospinal fluid; dsDNA, double-stranded DNA; DSS, dextran sulfate sodium; EAE, experimental autoimmune encephalomyelitis; EPC, endothelial progenitor cell; GCs, germinal centers; GD, Graves’ disease; IBD, inflammatory bowel disease; LN, lupus nephritis; MG, myasthenia gravis; MS, multiple sclerosis; NOD, non-obese diabetic; PBC, primary biliary cholangitis; pSS, primary Sjögren’s syndrome; RA, rheumatoid arthritis; SLE, systemic lupus erythematosus; SSc, systemic sclerosis; T1DM, type 1 diabetes; TGF-β1, transforming growth factor β1; Th1, T helper 1; Th17, T helper 17; Treg, regulatory T cell.

### CXCL13/CXCR5 Axis in Rheumatoid Arthritis

RA is a chronic inflammatory autoimmune disease characterized by inflammation of synovium and progressive destruction of joint, bone, and cartilage (113). B cells are critical contributors to RA, as they secrete proinflammatory cytokines, present antigens, and interact with other inflammatory cells (114). CXCL13, a regulator of B-cell homing and activation, is regarded as a novel biomarker of RA (115). Through single-cell RNA-seq analyses, Tph cells have been identified to be the predominant source of CXCL13 in RA synovial tissues, while CXCL13 expression was less detectable in synovial fibroblasts, vascular cells, macrophages, DCs, or other lymphocytes (116). Through secreting CXCL13, Tph cells can attract CXCR5^+^ cells towards inflamed synovium tissues (13). *In vitro* studies showed that Tph cells could induce the differentiation of memory B cells into plasma cells in IL-21 and signaling lymphocytic activation molecule (SLAM) family dependent manners (11). Furthermore, the frequency of Tph cells is robustly expanded in seropositive RA patients with higher disease activity, and is significantly declined after effective treatment, further supporting the pathogenetic role of these cells in RA (11).

Serum CXCL13 levels are found to be increased in RA patients, and are further elevated in rheumatoid factor (RF) positive and anti-citrullinated peptide antibodies (ACPA) positive patients (72). In both early and established RA cohorts, serum CXCL13 exhibits a strong correlation with serum IgM and IgA RF in seropositive RA patients (117). In early RA, baseline serum CXCL13 levels are correlated with elevated rates of joint damage during the 7-year follow-up period (118). Moreover, plasma CXCL13 concentrations are found to be higher in active RA patients when compared to quiescent RA or healthy controls. Moreover, plasma CXCL13 concentrations are positively correlated with clinical parameters, including C-reactive protein (CRP), erythrocyte sedimentation rate (ESR), RF, and the tender joint count in 68 joints (73). CXCL13 has also been detected in synovial tissues and synovial fluids (SF) in RA patients (63, 74). Furthermore, synovial CXCL13 expression is associated with markers that reflect immune cell activation and bone destruction in early RA (63). These results suggest that CXCL13 is a predictor of more severe and erosive RA (63). Studies exploring the possible association between CXCL13 levels and drug responsiveness have also been conducted. Notably, consistent results have also shown that the levels of CXCL13 in patient serum are markedly declined during therapy of disease modifying anti-rheumatic drugs (DMARDs) (119–121). In addition, higher baseline serum CXCL13 levels enable to predict faster return of circulating B cells following a course of rituximab, a B-cell depletion therapy (119). Collectively, these studies report the role of CXCL13 as a biomarker for treatment response in RA.

CXCL13 regulates lymphocyte aggregation and ectopic GCs formation in RA. CXCL13 and CCL20 synergistically drive the recruitment of B-cell towards inflammatory synovium of RA patients (122). Histological analysis indicates that CXCL13 is preferentially expressed in ectopic GCs within the RA synovium (65, 123). In addition, CXCL13 expression is associated with cluster enlargement and progressive organization of ELSs in arthritic joints, and has been identified to be an independent predictor for ectopic GCs formation (123, 124). Furthermore, in a rodent model of chronic antigen-induced arthritis, CXCR5^-/-^ mice showed impaired development and organization of ELSs along with alleviated joint destruction (125).

CXCL13 also drives the preferential migration and expression of IL-10 in B10^+^ cells, a subtype of regulatory B cells (Bregs) that plays an essential role in maintaining immune tolerance (75). However, it was found that CXCR5 expression was decreased in B10^+^ cells of RA patients, leading to impaired migration of B10^+^ cells toward CXCL13-rich synovial fluid, consistent with less IL-10 secretion (75).

In addition to the regulation of immune cells, CXCL13 also regulates endothelial progenitor cell (EPC) homing and angiogenesis during the development of RA (76). Blood-derived leukocytes enter into the synovial tissues through the vessels, which makes angiogenesis an important driver of RA progression (126). Tsai et al. found that CXCL13 promoted the expression of vascular endothelial growth factor (VEGF) in EPC *via* activation of PLC and MEK, with concomitant upregulated activator protein-1 (AP-1) signaling pathway, therefore contributing to EPC homing and angiogenesis during RA progression (76). Inhibition of CXCL13 using an short hairpin RNA (shRNA) in collagen-induced arthritis (CIA) mice markedly reduced EPC homing, angiogenesis, and arthritis severity (76).

Interestingly, antibodies against CXCL13 show beneficial effect in animal models of RA. In CIA mice, both prophylactic and therapeutic administration of CXCL13 neutralizing antibody curtailed the disease development, and alleviated the joint inflammation and cartilage damage (77). Anti-CXCL13 antibody reduced B cells in the lymphoid infiltration, and significantly attenuated cartilage damage and joint destruction (77, 127). Similarly, CXCR5-deficient mice showed severely impaired CIA, characterized by lower serum anti-collagen II antibody levels (78). In addition, selective ablation of CXCR5 expression in B cells or T cells also suppressed arthritis in CIA mice (78). Taken these studies into consideration, CXCL13/CXCR5 axis may be a novel treatment target for RA (57).

### CXCL13/CXCR5 Axis in Multiple Sclerosis

Multiple sclerosis (MS) is an autoimmune-mediated disease of the central nervous system (CNS), characterized by inflammation, demyelination, and neurodegeneration, which can lead to muscle weakness, visual dysfunction, sensory loss, ataxia, and cognitive impairment (128).

Increased CXCL13 concentrations have been detected in blood, cerebrospinal fluid (CSF), and actively demyelinating brain lesions in MS patients (79, 80). Notably, in patients with relapsing-remitting MS (RRMS), CSF CXCL13 levels are associated with increased relapse rate and disease severity measured by the expanded disability status scale (EDSS) (129). In addition, CSF CXCL13 levels are correlated with intrathecal immunoglobulin production, oligoclonal bands (OCBs), and the presence of lymphocytes (80, 130). In addition, CSF CXCL13 levels are reduced by treatment of methylprednisolone (131), daclizumab (132) and natalizumab (131, 133) in MS patients. Moreover, independent rituximab (134) and fingolimod therapy (135) also decreased CSF CXCL13 levels in RRMS. Collectively, CXCL13 is considered to be a potential marker for MS disease severity, prognosis, and clinical therapeutic responses.

The pathogenesis role of CXCL13 in MS is further demonstrated in experimental autoimmune encephalomyelitis (EAE) mice, a commonly used experimental animal model for human MS. Bagaeva et al. found that CXCL13^-/-^ mice showed a milder and self-limited form of EAE, as demonstrated by reduced clinical signs; attenuated inflammation, demyelination, gliosis, and fibrosis of white matter; and more complete recovery (81). However, the exact mechanisms of CXCL13/CXCR5 axis in MS are still unknown.

CXCL13 is involved in ELSs formation in MS, which may further lead to local humoral responses and inflammation. ELSs with a network of CXCL13^+^ cells, have been detected in the meninges of approximately 40% of secondary progressive MS (SPMS) patients, and are associated with more severe disease course and cortical lesions (69, 136–138). In addition, Columba-Cabezas et al. found that blocking the LT-LTβR signaling pathway in EAE mice inhibited CXCL13 production and ELSs formation in the meninges and suppressed EAE symptoms (139). Similarly, Pikor et al. demonstrated that CXCL13 was expressed by meningeal stromal cells in an LTβR dependent manner in a Th17 cell-driven EAE mice model (140). However, the predominant role of CXCL13 in MS is assumed to be attracting Tfh cells but not B cells. Quinn et al. explored the role of anti-CXCL13 antibody in EAE induced by adoptive transfer of myelin-specific Th17 cells (82). They found reduced Tfh cells infiltration in CNS, and decreased EAE severity, while infiltrated Th17 cells and B cells were largely unaffected (82). However, anti-CXCL13 antibody treatment was not effective in B-cell deficient EAE mice (82). Similarly, Rainey-Barger et al. demonstrated no difference in CNS B-cell infiltration between CXCL13^-/-^ mice and wild-type mice in actively induced EAE (141). However, the Th1 responses and Th17 responses were weakened two weeks after the peak of the disease in EAE mice with CXCL13 deficiency (141). These results suggest that CXCL13 may attracts Tfh cells toward the CNS, which further maintains immune responses mediated by B cells, Th1 cells, and Th17 cells in EAE. However, further studies are required to confirm this hypothesis in human MS.

### CXCL13/CXCR5 Axis in Systemic Lupus Erythematosus

SLE is a chronic autoimmune disease that affects multiple organs and tissues, such as the skin, joints, the CNS, and kidneys (142). Lupus nephritis (LN) is a glomerulonephritis caused by SLE, and can lead to irreversible kidney injury (143).

In the last couple of decades, increasing evidence has linked CXCL13/CXCR5 axis to SLE and its major manifestations. Serum CXCL13 levels are found to be significantly higher in SLE patients, especially in those with renal involvement, as compared with healthy controls (83, 84). Serum CXCL13 is positively correlated with the SLE Disease Activity Index (SLEDAI) (84, 144, 145), anti-double-stranded DNA (anti-dsDNA) antibodies titers, and prevalence of inflammatory arthritis, while it is inversely correlated with serum levels of complement factors C3 and C4 (146–148). CXCL13 is also a potential differentiation marker to identify active SLE from inactive SLE, and to identify LN from non-LN in SLE patients (148).

Expressions of CXCL13 and CXCR5 are also detected in the renal cortex from patients with LN (83). Renal CXCL13 mRNA levels were higher in LN patients with abundant intrarenal B cells infiltrate than those without (70). In addition, most infiltrating B cells express CXCR5 and are colocalized with CXCL13-abundant regions, suggesting that the CXCL13/CXCR5 axis plays a significant role in the recruitment of B cells in these inflammatory lesions (70). In addition to B-cell attraction, CXCL13 also induces double-negative (DN) T cells infiltration within inflammatory kidney tissues (85). In SLE, DN T cells could produce IgG anti-DNA antibody and inflammatory cytokines, and lead to tissues damages in the kidneys of SLE patients (149). In CXCR5^-/-^ B6/lpr mice, B-cell count and B-cell-mediated immune responses were diminished, and DN T cells accumulation was also reduced (85). B cells and DN T cells from CXCR5^-/-^ B6/lpr mice failed to migrate toward CXCL13 *in vitro* (85). Moreover, CXCL13 also regulates the balance between Th17 and regulatory T cells (Tregs) in SLE (86). In MRL/lpr lupus-prone mice, blockade of CXCL13 was found to significantly reduce spleen Th17/Tregs ratio, renal inflammatory cytokines production, and alleviated kidney damage (86).

Macrophages and renal DCs are reported to be the main sources of CXCL13 in lupus-prone mouse models (150, 151). In NZB/W F1 mice, a mouse model that resembles human SLE, elevated TLR7 and TLR9 responses induced increased CXCL13 expression in CD11b^+^ CD11^chi^ DCs, associated with dysregulated NF-κB signaling pathway (152). In addition, Moreth et al.’s study showed that elevated proteoglycan biglycan found in LN could trigger CXCL13 expression in macrophages and DCs in a TLR2- and TLR4-dependent manner (153). The CXCL13/CXCR5 axis also contributes to the proliferation of mesangial cells and to transforming growth factor-β1 (TGF-β1) production by activating ERK signaling pathway in LN. This process seems to be targeted by microRNA-155 (miR-155) (84, 87). An *in vitro* study found that CXCL13 induced secretion of proinflammatory cytokines and chemokines in human podocytes, *via* ERK signaling pathway (88). Furthermore, the cytokine and chemokine cocktail produced by activated podocytes leads to a respiratory burst in isolated human neutrophils (88). In addition, CXCL13 expression is significantly increased in circulating Tph cells and Tfh cells from SLE patients compared with controls (154). The frequency of Tph cells, but not Tfh cells, is positively correlated with both disease activity and CD11c^+^ B cells frequency in SLE patients (88). Tph cells may further support memory B-cell differentiation in SLE *via* IL-21 and MAF (88). However, further studies are needed to explore the regulatory roles of the CXCL13/CXCR5 axis in SLE, and whether this axis could be a novel target in human SLE also needs more evidence.

### CXCL13/CXCR5 Axis in Primary Sjögren’s Syndrome

The pSS is a chronic autoimmune disease characterized by lymphocytic infiltration within lacrimal and salivary glands, dryness of mouth and eyes, and elevated incidence of lymphoma (155). In pSS, dysregulated immune responses to the auto-antigens, such as Ro/SSA and La/SSB, result in epithelial destruction of the exocrine glands (155).

CXCL13 is proposed to be a biomarker of pSS severity. Serum CXCL13 levels are significantly elevated in pSS patients, and are found to correlate with clinical parameters, including rheumatoid factor (RF), κ-to-λ free light chain ratio, β2-microglobulin, γ-globulins, anti-Ro/SSA, anti-La/SSB, and erythrocyte sedimentation rate (ESR) (89, 90). Increased CXCL13 protein levels are also detected in pSS patients’ saliva (91), salivary glands tissues (92), and salivary gland secretome (i.e., salivary gland biopsy supernatants) (93). Interestingly, the serum concentrations of CXCL13 are also associated with the risk and occurrence of lymphoma in pSS patients (89, 90). Collectively, these observations highlight the potential role of CXCL13 in pSS.

CXCL13 is also described to be a biomarker of histological involvement in Sjögren’s syndrome. In pSS patients, CXCL13 is also associated with ELSs, a key mediator in pSS pathogenesis (156). Serum CXCL13 is associated with increased levels of lymphocytic infiltration, lymphoid organization, and the presence of ectopic GCs in pSS salivary glands (157). In pSS salivary gland tissues, CXCR5^+^ B cells are organized into GCs-like clusters (92). Moreover, Blokland et al. found a positive correlation between CXCL13 mRNA expression and Tfh cell frequencies in salivary gland of pSS patients (158). In addition, administration of abatacept, a recombinant fusion protein that selectively inhibited T-cell activation *via* binding to CD80 and CD86, significantly downregulated CXCL13 levels in pSS patients (159).

In a virus-induced murine model of lymphoid neogenesis in the salivary glands, IL-22 induced the expression of CXCL12 and CXCL13 in epithelial and fibroblastic stromal cells respectively, which is pivotal for B-cell recruitment, ELSs formation, and the aberrant autoimmune response (160).

CXCL13 neutralization results in a modest reduction of glandular inflammation (91). In addition, concomitant blockade of CXCL13 and B cell activating factor receptor (BAFFR) led to reduced salivary gland inflammation, total serum antibodies and anti-nuclear autoantibodies (ANA)-specific IgG autoantibody titers, along with preventing xerostomia development in a pSS mouse model (161). Recently, paeoniflorin-6’-O-benzene (CP-25) has been found to limit the target organ index and B cell activities in experiment Sjögren’s syndrome (ESS) by inhibiting CXCR5-G protein-coupled receptor kinase 2 (GRK2)-ERK/p38 signaling pathway (162). In addition, CP-25 also alleviated the salivary gland indexes; improved tissue integrity and saliva flow; and reduced the IgG antibodies, anti-SSA, and anti-SSB antibodies in ESS mice, by inhibiting JAK1-signal transducer and activator of transcription 1 and 2 (STAT1/2)-CXCL13 signaling and interfering with B-cell migration (163). These results suggest that the CXCL13/CXCR5 axis may serve as a new biomarker and a potential therapeutic target in pSS (91).

CXCL13 axis may also be implicated in the lacrimal gland pathology in pSS. LTβR blockade in the Non-Obese Diabetic (NOD) model of Sjögren’s syndrome significantly reduced CXCL13 protein levels in lacrimal glands, accompanied by reduced B-cell accumulation, eliminated high endothelial venule (HEV) in lacrimal glands, reduced entry rate of lymphocytes into lacrimal glands, and improved tear-fluid secretion and corneal integrity (164). However, further studies are required to elucidate the role of the CXCL13/CXCR5 axis in keratoconjunctivitis sicca of human pSS.

### CXCL13/CXCR5 Axis in Myasthenia Gravis

Myasthenia gravis (MG) is an autoimmune disease caused by autoantibodies against acetylcholine receptors (AChR) or other functionally related molecules in the postsynaptic membrane at the neuromuscular junction (165). Muscle weakness is the prominent feature of MG, and typically develops with repetitive muscle use and as the day progresses (166).

Thymic lymphoid hyperplasia, characterized by the presence of ectopic GCs, has been found in most of the patients with early-onset MG (165). Thymic CXCL13 expression levels are significantly elevated, and are correlated with disease severity in MG patients with thymic hyperplasia (71, 94–97). Furthermore, serum CXCL13 levels are increased in MG patients (95, 96, 98), and are strongly correlated with disease severity and the frequency of circulating Tfh cells (98). In addition, corticosteroid treatment markedly reduced thymic CXCL13 expression and GCs formation in MG patients (95). Moreover, DNA microarray analysis showed that CXCL13 was the gene on which glucocorticoids exert the most significant effect, suggesting that CXCL13 is a major target of glucocorticoids in MG treatment (95).

CXCL13 is a target gene of miR-143, a tumor suppressor microRNA (167). MiR-143 can repress proliferation and enhance apoptosis of thymocytes through negatively targeting CXCL13 (167). CXCL13 is also negatively regulated by miR-548k (168). Downregulated expression of miR-548k may contribute to elevated CXCL13 levels in thymus of MG patients with thymus hyperplasia (168).

CXCL13 is constitutively produced by thymic epithelial cells (TECs) in the normal thymus and is overexpressed in MG TECs, which may contribute to the elevated CXCL13 levels in MG thymus (95). Polyinosinic-polycytidylic acid (Poly(I:C)) injection triggers a local increase in type I interferons (IFN-I) in mouse thymus, associated with overexpression of CXCL13, CCL21, BAFF, and increased recruitment of B cells (169). In addition, an *in vitro* study showed that IFN-β induced CXCL13 and CCL21 expressions in TECs and lymphatic endothelial cells respectively (169). These results suggest that IFN-I can trigger chemokine expressions that favor ectopic GCs development in MG thymus (169). However, in a transgenic mouse model with overexpressed CXCL13 in the thymus, thymic CXCL13 overexpression by itself is not sufficient to induce B-cell recruitment (66). Interestingly, under the inflammatory conditions induced by Poly(I:C), this thymic CXCL13 overexpression model gets a strong recruitment of B cells to the thymus, and is more susceptible to developing MG, as demonstrated by elevated clinical signs, anti-AChR antibody levels, and thymic GCs-like structures, when compared with wild-type mice (66). These observations suggest that CXCL13 can exaggerate the pathogenesis of MG, rather than initiating the onset of the disease.

A subtle dual-regulated effect of estrogen has been found on CXCL13 expression in MG (170). In the resting state, estrogen inhibits CXCL13 expression of TECs (170). However, under inflammatory conditions, the direct inhibited effect of estrogen is overpassed by inflammatory pathways, such as TLR/IFN-I related pathways (170). Consequently, estrogen contributes to the sustained activation of these pathways and promotes the expression levels of some chemokines including CXCL13 (170).

### CXCL13/CXCR5 Axis in Other Autoimmune Diseases

Emerging studies have also found that CXCL13 can be served as a potential biomarker for other autoimmune diseases and autoimmune-related disorders such as type 1 diabetes mellitus (T1DM), inflammatory bowel disease (IBD), primary biliary cholangitis (PBC), Graves’ disease (GD), bullous pemphigoid (BP), psoriasis, systemic sclerosis (SSc), autoimmune pancreatitis (AIP), and common variable immunodeficiency (CVID).

T1DM is a chronic autoimmune disease that results from pancreatic islet β-cells destruction, leading to insulin deficiency and hyperglycemia (171). Several studies conducted on animal models aimed to elucidate the role of CXCL13 in T1DM. However, the results seem to be equivocal. The initial study utilized transgenic mice that expressed CXCL13 in islet β-cells, and found B-cell infiltration within the islets of these mice (99). Furthermore, the study also found the presence of well-characterized ELSs in large-sized infiltration within the pancreas of these transgenic mice (99). However, this model failed to mimic a diabetes phenotype of impaired β-cells and increased blood glucose (99). In the autoimmune Non-Obese Diabetic (NOD) T1DM mouse model, CXCL13 and its cognate receptor, CXCR5, were highly expressed during the development of ELSs, especially in the early onset of diabetes (100). In addition, a high expression of CXCR5 was also observed in islet-infiltrating B220^+^ cells (100). In NOD mice, CXCL13 blockade disrupted B-cell organization in islet ELSs, however, neither the proportion of B-cell within the islets, nor the T1DM disease progression were affected (172). These results suggest that CXCL13 plays role in the neogenesis and maintenance of ELSs in islets of T1DM, but whether CXCL13 is involved in the development of T1DM remains to be further studied. In addition, Vecchione et al. evaluated plasma CXCL13 levels of children with new-onset T1DM, autoantibody-positive at-risk children, and autoantibody-negative control children (173). However, the results showed no significant difference in plasma CXCL13 levels among the three groups (173). In a recent study of Vecchione et al., plasma CXCL13 levels were evaluated in T1DM, autoantibody-positive, and autoantibody-negative adult subjects (174). The study found that plasma CXCL13 levels in T1DM subjects were significantly decreased compared to non-diabetic autoantibody-negative and autoantibody-positive at-risk subjects (174). Thus, the role of CXCL13 in human T1DM also needs to be further studied to take further conclusions.

IBD, mainly comprised of Crohn’s disease and ulcerative colitis (UC), are chronic inflammatory disorders of the gastrointestinal tract (175). Although the exact etiology of IBD remains unknown, it involves a complex interaction between the genetic, environmental, epithelial, microbial, and immune factors (175). Circulating CXCL13 levels are increased in both pediatric (101) and adult (102) patients with IBD. Abundant expressions of CXCL13 and CXCR5 were also detected within aberrant lymphoid aggregates in UC lesions (103). In addition, CXCR5 gene polymorphisms can influence IBD susceptibility. The CXCR5 rs6421571 allele C is associated with increased risks of Crohn’s disease in the Japanese population (176). These results support the possible involvement of CXCL13 and CXCR5 in IBD. Monocytes/macrophages seem to be the main producers of CXCL13 in inflammatory UC lesions where lymphoid neogenesis occurs (177). However, in a UC mice model induced by dextran sulfate sodium (DSS), CXCL13 is produced by GP38^+^ colonic stromal cells, mediated by the innervation of the vagus nerve (178). Selective surgical ablation of vagus nerve innervation inhibited local CXCL13 expression and abrogated ELSs formation but did not affect colitis (178). In addition, in the DSS-induced UC mice model, the OX40/OX40L axis can induce CXCR5 expression on CD4^+^ T cells, and further promote their migration toward GCs in the lesioned colonic mucosa (179). Anti-CXCL13 antibody treatment reduced the disease severity in the DSS-induced UC, suggesting CXCL13 as a potential target for treating UC (101).

PBC is the prototype of an autoimmune disease characterized by destructive lymphocytic cholangitis and the presence of serum anti-mitochondrial antibodies (AMAs) targeting at the E2 subunit of the pyruvate dehydrogenase complex (PDC-E2) (180). Serum CXCL13 is increased in patients with PBC, and is associated with total bilirubin levels, and is gradually decreased during the longitudinal phase of ursodeoxycholic acid treatment (104). Elevated CXCL13 expression was also found in liver tissues of PBC patients, especially in portal tracts areas (104). In addition, expanded CXCR5^+^ CD4^+^ T cells, CD19^+^ B cells, and CD19^+^ CD38^+^ B cells were detected in portal tracts of PBC, accompanied with increased expression of intrahepatic IL-21 (104). These results suggest that CXCL13 may serve as a key regulator for B-cell dysregulation in PBC. In addition, genome-wide association studies have identified CXCR5 (rs6421571) as a risk locus for PBC in both UK and Japanese populations (176, 181).

GD is an autoimmune thyroid disease characterized by the presence of autoantibodies against the thyroid-stimulating hormone receptor (TSH-R), thyroglobulin and thyroperoxidase (182). CXCL13 and CXCR5 expressions is increased in GD thyroid tissues (105–107). CXCL13 expression levels are significantly associated with focal lymphocytic infiltrates, the presence of ectopic GCs, and anti-thyroperoxidase autoantibodies (105, 106). The above-mentioned evidence suggests that dysregulated CXCL13 expression is related to the pathogenesis of GD.

BP is the most common type of pemphigoid diseases which is characterized by autoantibodies against structural proteins of the hemidesmosomes, leading to tense blisters and erosions on the skin or mucous membranes (183). Serum CXCL13 levels and the proportion of CXCL13^+^ cells among leukocytes in lesioned skin are both elevated in patients with BP, and are positively correlated with anti-BP180-NC16A titers in BP patients (108).

Psoriasis is a chronic immune-mediated inflammatory skin disease, characterized by clinical features of erythema, thickening, and scale (184). CXCL13 circulating levels and CXCL13 expression levels in cutaneous lesions are increased in psoriasis, and are positively correlated with disease severity (109, 110). Moreover, CXCL13 expression levels in psoriatic lesions are decreased after anti-IL23 treatment (109). Plasma CXCL13 levels are positively correlated with the frequency of peripheral helper T 17 (Tph17) cells and Tfh cells in peripheral blood of psoriasis patients (110). Additionally, subsets of CD8^+^ T cells, characterized by expression of IL-17 pathway cytokines, cytolytic genes, and CXCL13, were specifically detected in psoriasis lesions (109).

SSc, also known as scleroderma, is characterized by immune dysregulation, fibrosis of the skin and internal organs, and vasculopathy (185). Serum CXCL13 levels are increased in patients with SSc compared with healthy controls (111). Notably, serum CXCL13 levels are associated with tissue fibrosis, vasculopathy and immune activation, especially with interstitial lung disease and digital ulcers in SSc patients (111). Recently, Gaydosik et al. identified a cluster of recirculating CXCL13^+^ T cells, characterized by a Tfh-like gene expression profile, which may promote B-cell responses within the lesioned skin in SSc (186).

AIP is the pancreatic manifestation of systemic IgG4-related disease (IgG4-RD), characterized by increased serum IgG4 levels and lymphoplasmacytic sclerosing pancreatitis (187). In patients with AIP, mRNA levels of CXCL13, consistent with CCL19, CCL21, CCL17, BAFF, LT-α, and LT-β expression were increased in pancreatic tissues, compared with controls (112). In addition, serum levels of CXCL13, CCL19, TNF-α, and IL-6 were also elevated in AIP patients (112). Overexpression of LT-α, and LT-β in pancreatic acinar cells of mice induced CXCL13 expression and other features resembling human AIP (112). Furthermore, these features were significant ameliorated through inhibiting LTβR-signaling (112). Therefore, LT-LTβR axis plays a pivotal role in AIP by mediating chemokines and pro-inflammatory cytokines such as CXCL13. At present, there is still a lack of study on the role of CXCL13 in other types of IgG4-RD such as orbital disease, sclerosing cholangitis, retroperitoneal fibrosis, and interstitial nephritis, and more attention is needed to address this issue.

CXCL13 is also associated with immunodeficiency associated autoimmune diseases such as CVID. CVID is a heterogeneous group of primary immunodeficiency diseases characterized by hypogammaglobinemia (188). Patients with CVID are often complicated with respiratory tract infection, inflammatory diseases, and autoimmune diseases (188). Hultberg et al. evaluated plasma protein profiles of patients with CVID through proximity extension assay, and found that CXCL13 was the top upregulated extracellular protein in CVID, as compared with healthy controls (189). Due to the heterogeneity of CVID, the role of CXCL13 in the disease still needs to be further studied.

However, there has been only limited research on the function of CXCL13/CXCR5 axis in these autoimmune diseases, and more studies are required to provide further insights.

## CXCL13/CXCR5 Axis as a Potential Therapeutic for Autoimmune Diseases

Due to its essential role in immune regulation and inflammatory response, CXCL13/CXCR5 axis may serve as a potential therapeutic target for autoimmune diseases (**Table 2**). Indeed, numerous preclinical studies support the concept of suppressing the CXCL13/CXCR5 axis as a novel therapeutic approach for autoimmune diseases treatment. For example, in CIA mice, prophylactic or therapeutic administration of anti-CXCL13 monoclonal antibody curtailed the disease development, consistent with alleviated joint inflammation and cartilage damage (77). In addition, Tsai et al. reported that inhibition of CXCL13 action through an shRNA markedly reduced angiogenesis and arthritis severity in CIA mice (76). Moreover, several studies have revealed that CXCL13 neutralization by anti-CXCL13 antibody also attenuates disease progression and severity in EAE mice (77, 81, 82). To assess the role of CXCL13 in LN, Wu et al. used neutralizing anti-CXCL13 antibodies to block MRL/lpr lupus-prone mice (86). The results showed that CXCL13 neutralization significantly improved kidney injury, and reduced serum anti-dsDNA titers, renal immune complex deposition, renal inflammatory cytokines production, and spleen Th17/Tregs ratio (86). Similarly, in a mouse model of pSS, CXCL13 neutralization by anti-CXCL13 monoclonal antibody results in a modest reduction of glandular inflammation (91). In addition, in a UC mice model induced by DSS, anti-CXCL13 antibody significantly reduced the disease severity (101). To summarize, these results suggest that the CXCL13/CXCR5 axis is a promising target for autoimmune diseases, which may open avenues for novel strategies for autoimmune diseases treatment.

**Table 2:** Therapeutic effects on targeting CXCL13/CXCR5 axis in autoimmune diseases.

Reagent	Biological source	Animal model	Disease	Effects	Reference
Anti-CXCL13 Abs	Goat	CIA in DBA/1 mice	RA	Decreased arthritis severity and lymphoid neogenesis in joints.	(127)
Anti-mouse CXCL13 Abs	NA	Adoptively transferred EAE in C57BL/6 mice	MS	Attenuated EAE clinical scores and reduced inflammatory demyelinated lesions in spinal cords.	(82)
Anti-human CXCL13 mAb, MAB5378	Mouse	CIA in DBA1/J mice	RA	Delayed arthritis development and reduced arthritis severity.	(77)
Anti-human CXCL13 mAb, MAB5378	Mouse	Adoptively transferred EAE in SJL/J mice	MS	Decreased clinical severity of Th17-induced EAE but not Th1-induced EAE.	(77)
Anti-human CXCL13 mAb, MAB5378	Mouse	Actively induced EAE in SJL/J mice	MS	Decreased EAE clinical severity.	(77)
Anti-human CXCL13 mAb, MAB5378	Mouse	NOD/ShiLtJ mice	pSS	Reduced lymphocytic foci within submandibular gland.	(91)
Anti-mouse CXCL13 mAb, MAB470	Rat	Adoptively transferred EAE in B10.PL mice	MS	Decreased EAE clinical scores.	(81)
Anti-mouse CXCL13 mAb, MAB470	Rat	NOD/ShiLtJ mice	T1DM	Disrupted B-cell organization in islet but did not affect disease development.	(172)
Anti-mouse CXCL13 mAb, MAB470	Rat	DSS-induced UC in C57BL/6 mice	IBD	Increased colonic length and decreased colitis severity.	(101)
Anti-mouse CXCL13 mAb, MAB4701	Rat	MRL/lpr mice	SLE	Attenuate kidney injury; reduced serum anti-dsDNA titres, renal immune complex deposition, renal inflammatory cytokines expression, and spleen Th17/Tregs ratio.	(86)
shRNA targeting CXCL13	NA	CIA in C57BL/6J mice	RA	Mitigated arthritis activity; reduced VEGF expression, EPC homing, and angiogenesis in joints.	(76)
Paeoniflorin-6’-O-benzene sulfonate (CP-25)	NA	ESS in C57BL/6 mice	pSS	Improved saliva flow and alleviated histopathology of submandibular gland through targeting JAK1-STAT1/2-CXCL13 and CXCR5-GRK2-ERK/p38 signaling pathways.	(162, 163)

Abs, autoantibodies; CIA, collagen-induced arthritis; dsDNA, double-stranded DNA; DSS, dextran sulfate sodium; EAE, experimental autoimmune encephalomyelitis; ESS, sxperimental Sjögren’s syndrome; EPC, endothelial progenitor cell; ERK, extracellular signal-regulated kinase; GRK, G protein-coupled receptor kinase; IBD, inflammatory bowel disease; JAK, Janus kinase; mAb, monoclonal antibody; MS, multiple sclerosis; NA, not available; NOD, non-obese diabetic; pSS, primary Sjögren’s syndrome; RA, rheumatoid arthritis; shRNA, short hairpin RNA; SLE, systemic lupus erythematosus; STAT, signal transducer and activator of transcription; T1DM, type 1 diabetes; Th17, T helper 17; Tregs, regulatory T cells; UC, ulcerative colitis; VEGF, vascular endothelial growth factor

## Discussion

This review highlights the biological functions, protein structures, signaling transduction pathways, and roles of CXCL13/CXCR5 in the pathogenesis of autoimmune diseases. Autoimmune diseases are characterized by immune dysfunction, autoantibody production and chronic inflammation. In this context, the role of CXCL13 as a B-cell chemokine and key regulator of humoral immunity has attracted considerable attention in the study of the development of autoimmune diseases.

Abnormal expression of CXCL13, consistent with that of other chemokines and cytokines, contributes to ectopic lymphoid neogenesis in local lesioned tissues, and promotes the production of autoantibodies in autoimmunity (62). In addition, recent studies have revealed that CXCL13/CXCR5 may also have a disease-specific pathological mechanism in some autoimmune diseases. For example, in RA, CXCL13 regulates EPC, thereby promoting angiogenesis (76). In LN, CXCL13 regulates DN T cells, mesangial cells and podocytes and leads to renal damage (85, 87, 88). However, the specific mechanism through which CXCL13 plays a role in autoimmune diseases is still largely unknown. It is necessary to utilize better animal models, human pathological specimens, and *in vitro* research to further investigate the regulatory role of CXCL13 in ectopic lymphoid neogenesis, immune cell activation and other key pathological processes in autoimmunity. In addition to autoimmune diseases, CXCL13 is also regarded as a prognostic biomarker for IPF (27, 190). CXCL13 was overexpressed in lung tissues and plasma of patients with IPF (27). Plasma CXCL13 levels were positively correlated with disease manifestations and prognoses of IPF patients (27). During *Pneumocystis* infection, CXCL13 was required for the formation of inducible bronchus associated lymphoid tissues (iBALT), the ELSs in the lung (191). In *Pneumocystis*-infected Rag2^−/−^ Il2rg^−/−^ mouse, the expression of CXCL13 was increased (191). In addition, CXCR5^-/-^ mice lacked draining LNs and presented poorly organized iBALT structures in the lung after *Pneumocystis* infection (191). CXCL13 can be produced by pulmonary macrophages under the stimulation of TNF-α, IL-10 (192), and can also be produced by pulmonary fibroblasts under the stimulation of IL-13 and IL-17A (191). In activated alveolar macrophages (AM) and monocyte-derived macrophages (MoDM), the CXCL13 expression was induced by NF-κB and JAK/STAT pathways (192). Besides, both IL-13 and IL-17A synergistically upregulated CXCL13 expression in pulmonary fibroblasts in STAT3- and GATA3-dependent manner (191). 

Clinical studies have shown that CXCL13 expression is elevated in autoimmune diseases patients, and is correlated with clinical parameters that are related to disease severity, activity, and prognosis (73, 129, 193–195). In addition, in the clinical setting, glucocorticoid and DMARDs can alleviate the symptoms of autoimmune diseases and reduce the expression of CXCL13, suggesting that CXCL13 may reflect treatment response (95, 134). However, due to the heterogeneity of autoimmune diseases, the clinical significance of CXCL13 may be different for patients with different disease subtypes, different stages of progression, and different genetic backgrounds. Further studies are required to explore this issue. Moreover, CXCL13 combined with other biomarkers may be helpful for the diagnosis of some autoimmune diseases, but the optimal cut-off value should be determined according to specificity and sensitivity, and their diagnostic efficacy should be carefully evaluated.

In animal models of autoimmune diseases, knockout or neutralization of CXCL13/CXCR5 significantly improve clinical symptoms, suggesting that CXCL13/CXCR5 can be used as a therapeutic target for autoimmune diseases (77). However, due to the gap between animal models and human diseases, further studies are required to assess if this therapeutic effect can be reflected in humans and whether the drugs targeting CXCL13/CXCR5 are safe. Contrastingly, neutralizing antibodies are the most frequently used methods to target CXCL13/CXCR5, although there is still a lack of studies on RNA interference (RNAi) or small molecule inhibitors targeting CXCL13/CXCR5. The development of highly selective and stable small molecule inhibitors targeting CXCL13/CXCR5 will become a focus of future research. In addition, the protein structure of CXCR5 is still largely unknown, and analysis of the protein structure of CXCR5 will accelerate the development of relevant drugs. In brief, although numerous challenges remain, CXCL13/CXCR5 axis is undoubtedly a promising therapeutic target for human autoimmune diseases.

## Author Contributions

ZP, TZ, and YL responsible for literature research and writing. NZ reviewed the manuscript, made significant revisions on the drafts, and supervised and finalized this work. All authors have read and agreed to the published version of the manuscript.

## Funding

The research was supported by National Natural Science Foundation of China (No.81970738 and No.81600157), Key Research and Development Program of Sichuan Province (No. 2020YFS0071), and Universal Application Program of Health Commission of Sichuan Province (No.21PJ047).

## Conflict of Interest

The authors declare that the research was conducted in the absence of any commercial or financial relationships that could be construed as a potential conflict of interest.

## Publisher’s Note

All claims expressed in this article are solely those of the authors and do not necessarily represent those of their affiliated organizations, or those of the publisher, the editors and the reviewers. Any product that may be evaluated in this article, or claim that may be made by its manufacturer, is not guaranteed or endorsed by the publisher.
