# Corrigendum: Role of the CXCL13/CXCR5 axis in autoimmune diseases

**DOI:** 10.3389/fimmu.2022.1061939

**Published:** 2022-10-20

**Authors:** Zijian Pan, Tong Zhu, Yanjun Liu, Nannan Zhang

**Affiliations:** ^1^ National Center for Birth Defect Monitoring, Key Laboratory of Birth Defects and Related Diseases of Women and Children, Ministry of Education, West China Second University Hospital, and State Key Laboratory of Oral Diseases, Sichuan University, Chengdu, China; ^2^ West China Hospital of Stomatology, Sichuan University, Chengdu, China

**Keywords:** CXCL13, CXCR5, chemokine, autoimmunity, therapeutic target

There were errors in the body text of our original article. In the **CXCL13/CXCR5 Protein Structure** section, we unintentionally misinterpreted the cited article by Rosenberg et al., entitled “The N-terminal length and side-chain composition of CXCL13 affect crystallization, structure and functional activity”, and erroneously confused the protein structure of “Met CXCL13” with “wild type (WT) CXCL13”. In addition, we also mistakenly overlooked the presence of the signal peptide in the CXCL13 precursor.

A correction has been made to **CXCL13/CXCR5 Protein Structure**:

“CXCL13 is a 109-amino-acid protein with a signal peptide of 22 amino acids, containing four cysteine residues showing a C-X-C chemokine pattern (**Figure 3A**) (4). Typically, the tertiary structure of chemokines is relatively conserved, consisting of a disordered N-terminal ‘signaling domain’, followed by a ‘core domain’, which includes an ‘N-loop’, a 3_10_-helix, a three-stranded β-sheet, and a C-terminal α-helix (44). Although the crystal structure of wild-type (WT) human CXCL13 protein alone has not been solved yet, Tu et al. crystallized two structures of WT human CXCL13 in complex with antibody single chain variable fragments (scFvs) (45). Moreover, Rosenberg et al. solved the structures of two engineered CXCL13 mutants, *i.e.* Met CXCL13 (mature CXCL13 protein contains a N-terminal initiating methionine) and Δ1L2M CXCL13 (mature CXCL13 protein in which Val1 is deleted and Leu2 is replaced by Met) (46). In Met CXCL13, the N-terminus forms an additional parallel β-strand (β0), which interacts with the core β-sheet, thus forming a four-stranded β-sheet (**Figure 3B**). A β-strand (β-1) in the N-terminus is also evident in Δ1L2M CXCL13 (**Figure 3C**), and it interacts with both the core β-sheet from an adjacent monomer and a β-strand (β0) formed by the N-loop in the same monomer. These structures, together with the structures of CXCL13 in complex with scFvs demonstrate a flexible N-terminus, as well as a highly disordered C-terminal extension, but a fairly rigid canonical chemokine core domain of CXCL13.”

**Figure 3:**
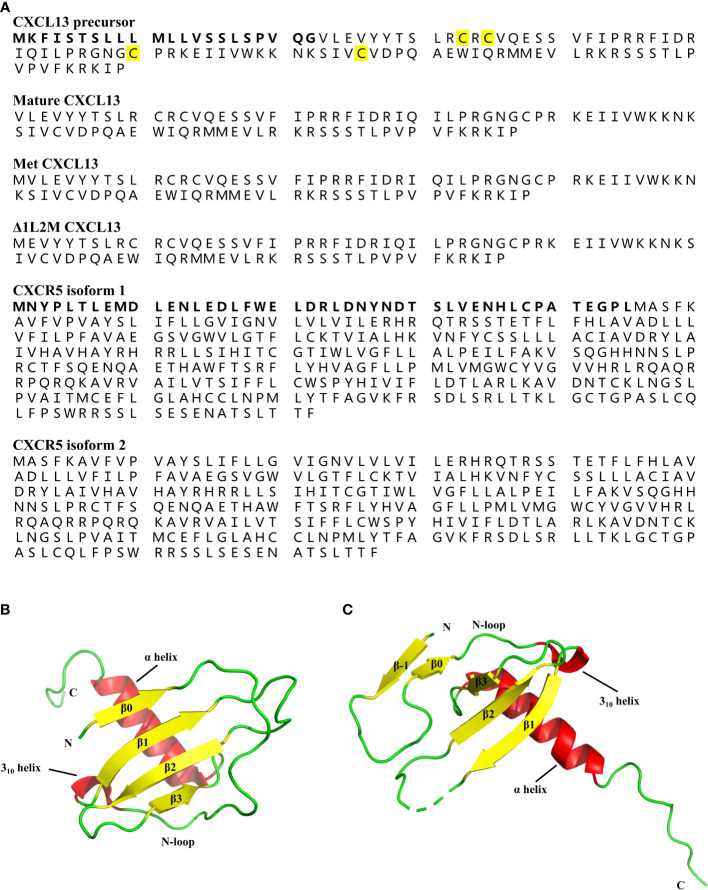
Protein structures of CXCL13 and CXCR5. **(A)** The amino acid sequence of human CXCL13 precursor, mature CXCL13, Met CXCL13, Δ1L2M CXCL13, and two isoforms of human CXCR5. The human CXCL13 protein consists of 109 amino acids, including a signal peptide of 22 amino acids (marked in bold). The four conserved cysteine residues are marked in yellow. The human CXCR5 has two isoforms due to the alternatively spliced transcript variants. The difference between the two isoform of CXCR5 is additionally marked in bold. **(B)** The tertiary structure of Met CXCL13 [Protein Data Bank (PDB) ID: 7JNY]. The N-terminus of Met CXCL13 forms a β0-sheet, followed by a long N-loop ending in a short 3_10_-helix, and the central three-stranded anti-parallel β-sheet, and a C-terminal α-helix. β-sheet is indicated by yellow arrows, and α-helix and 3_10_-helix are indicated by red cylinders. **(C)** The tertiary structure of Δ1L2M CXCL13 monomer [PDB ID: 6VGJ]. In Δ1L2M CXCL13, the N-terminus folds into a β-strand (β-1), followed by a β0-sheet, and a canonical chemokine core domain. β-sheet is indicated by yellow arrows, and α-helix and 3_10_-helix are indicated by red cylinders.

Accordingly, a correction has been made to **Figure 3** and corresponding figure legend.

The authors apologize for these errors and state that this does not change the scientific conclusions of the article in any way. The original article has been updated.

## Publisher’s note

All claims expressed in this article are solely those of the authors and do not necessarily represent those of their affiliated organizations, or those of the publisher, the editors and the reviewers. Any product that may be evaluated in this article, or claim that may be made by its manufacturer, is not guaranteed or endorsed by the publisher.

